# Development of a Wheel-Type In-Pipe Robot Using Continuously Variable Transmission Mechanisms for Pipeline Inspection

**DOI:** 10.3390/biomimetics9020113

**Published:** 2024-02-14

**Authors:** Jeongyeol Park, Tuan Luong, Hyungpil Moon

**Affiliations:** Department of Mechanical Engineering, Sungkyunkwan University, Suwon 16419, Republic of Korea; flashpark@g.skku.edu

**Keywords:** in-pipe robot, CVT mechanism, wheel-type robot, bio-inspired robot

## Abstract

Pipelines are embedded in industrial sites and residential environments, and maintaining these pipes is crucial to prevent leakage. Given that most pipelines are buried, the development of robots capable of exploring their interiors is essential. In this work, we introduce a novel in-pipe robot utilizing Continuously Variable Transmission (CVT) mechanisms for navigating various pipes, including vertical and curved pipes. The robot comprises one air motor, three CVT mechanisms, and six wheels at the end of six slider-crank mechanisms, including three active and three idler ones. The slider crank and spring mechanism generate a wall press force through the wheel to prevent slipping inside the pipe. This capability allows the robot to climb vertical pipes and adapt to various pipe diameters. Moreover, by combining CVT mechanisms, whose speed ratios between the driver and driven pulleys are passively adjusted by the position of the slider, the robot achieves independent and continuous speed control for each wheel. This enables it to navigate pipes with various geometries, such as straight–curved–straight pipes, using only one motor. Since active control of each wheel is not needed, the complexities of the robot controller can be significantly reduced. To validate the proposed mechanism, MATLAB simulations were conducted, and in-pipe driving experiments were executed. Both simulation and experimental results have shown that the robot can effectively navigate curved pipes with a maximum speed of 17.5 mm/s and a maximum traction force of 56.84 N.

## 1. Introduction

As pipelines are used in various industrial sites and residential areas, the maintenance of pipes is essential to prevent accidents and economic losses caused by the aging of pipes. While externally exposed pipelines are inspected by humans themselves or by out-pipe robots such as [1,2], many pipelines are buried in the ground or on the inner walls of buildings. Since these pipes are difficult to inspect from the outside, in-pipe robots that can drive inside the pipe have been developed.

In-pipe robots can be classified based on locomotion, actuator, and power supply, as illustrated in Table 1. Regarding actuators, common types include pneumatic actuators [3,4], DC motors [5], and new artificial actuators like Shape memory alloy actuators (SMAs) [6]. In terms of power supply, in-pipe robots are powered through cable [3] or battery [5]. Additionally, in-pipe robots can be categorized into active and passive locomotion. Robots with passive locomotion are propelled by the liquid flow inside the pipe and are typically equipped with pressure inspection gauge (PIG) mechanisms [7]. In-pipe robots with active locomotion are further classified into wheeled types [8,9,10,11], Caterpillar type [12,13], and non-wheeled types [14,15].

Wheel-driven pipe-inspection robots are among the most common types and can be further divided into three subcategories: simple-structure robots, wall-press robots, and screw drive robots. Simple structure robots have wheels connected under their body, similar to regular wheeled robots [16]. Wall-press robots employ wheel modules positioned at an angle with each other, continuously pressing against the inner surface of the pipe [17]. In screw drive types, the wheels are mounted on a rotational unit and a fixed unit, transmitting the helical motion of the wheels to linear motion along the pipe axis [11]. The Caterpillar-type robot can also be classified into simple structure and wall-press types [18,19]. In comparison to wheel-type robots, the Caterpillar-type ensures reliable motion due to the high friction generated between the robot and the pipe, achieved through the use of belts and wheels. In the non-wheeled types, inchworm-type robots [3] achieve movement inside pipes through the contraction and extension of a flexible body along the pipe axis. On the other hand, snake-type robots [20] are composed of modular units that enable motion inspired by the serpentine locomotion of a snake. Leg-type robots utilize legs or arms for internal pipe inspection, demonstrating notable capability in complicated configurations, although they require sophisticated algorithms [21,22,23]. For small-diameter pipes, free-swimming robots are equipped with propellers so that the robot can move inside the pipe using propulsion force [24]. Comparisons between different in-pipe robots can be found in Table 2. A comprehensive review of in-pipe robots can be found in Table 2. A comprehensive review of in-pipe robots can be found in [25].

**Table 1:** Categorization of in-pipe robots (adapted from [25]).

Actuator	Pneumatic Actuators		
	**DC Motors**		
	**SMA**		
**Power Supply**	**Cable**		
	**Battery**		
Locomotion	Active locomotion	Wheel type	Simple structure
			Wall-press
			screw-drive
		Caterpillar type	Simple structure
			Wall-press
		Non-wheeled type	Inchworm
			legged
			Free swimming
	Passive locomotion	PIG	

While various types of in-pipe robots have been developed, each possesses its own set of advantages and disadvantages. Continuous advancements in in-pipe robot technologies remain crucial to optimize performance in terms of mobility, adaptability, and driving force across different pipe types and environments. In terms of pipe types, some common challenging ones are pipes with variable diameters, vertical pipes, and pipes with complex inner geometries, such as elbow, miter, and branch pipes. To address these challenges, robots need mechanisms that enable adaptation to variations in pipe dimensions and the ability to adjust speed accordingly. Another challenge appears when the in-pipe robots are working in in-service networks [26,27]. In this case, the presence of flow, such as water and gases, will execute drag forces on the robot. This problem might be addressed using control or design solutions. In [26], the fluid flow was considered a disturbance and was overcome by a nonlinear sliding mode controller. A design solution has been presented in [27], in which computational fluid dynamics computation was carried out to simulate the effect of different conditions of the water flow (pressure, flow velocity) on the in-pipe robot. The gear motors were selected to provide equal traction force to overcome the maximum drag force.

In this work, we present an initial effort to create a new in-pipe inspection robot, specifically a wall-push wheel type designed to climb vertical and curved pipes with a target size of 150 mm and without the presence of fluid flow. Our contribution includes the integration of slider-crank mechanisms and continuously variable transmission (CVT) mechanisms, which enables the robot to traverse pipes with variable diameters without the need for pipe geometry recognition or active control of the wheels. It is worth noting that the velocities of the three active wheels can be modulated independently using only one motor, a notable contrast to the use of three motors, as seen in [18]. While the continuously variable transmission (CVT) mechanism has been utilized to enhance energy transmission efficiency [28,29] and speed control of holonomic vehicles [30], to the best of the author’s knowledge, our current proposal represents the first instance of its application to achieve speed variation in an in-pipe robot.

## 2. Robot Design

### 2.1. Design Objectives

**Figure 1:**
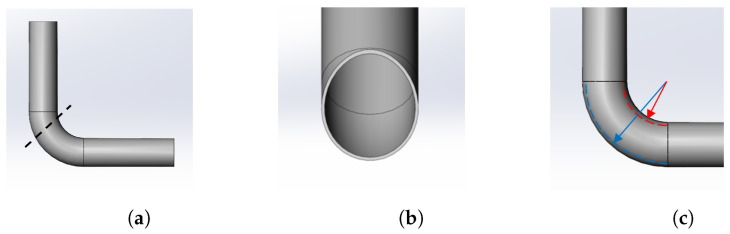
Schematic of the target curved pipe: (**a**) the cutting line intersects the curved part of the pipe, (**b**) the cross-section of the pipe at the cutting line exhibits an egg-like shape rather than a circular one, and (**c**) as a result, different velocities for different wheels are necessary when passing through the curved pipe due to varying turning radii. (**a**) A 3D view of curved pipe and the cutting line; (**b**) cross-section of the pipe at the cutting line; (**c**) different turning radii in the curved pipe.

The design objectives of our robot are to navigate vertical and curved pipes with a target diameter of 150 mm, in the absence of fluid flows such as water or gases. When climbing a vertical pipe, the weight of the air motor is taken into account to estimate the required traction force. In the case of a curved pipe, each wheel must have the capability to independently change its position and velocity, considering the different turning radius of each wheel during the passage, as illustrated in Figure 1.

### 2.2. Overview of Robot

In this section, we provide an overview of our robot’s configuration, offering insights into its design and functionality that contribute to its performance to achieve the design objectives.

**Figure 2:**
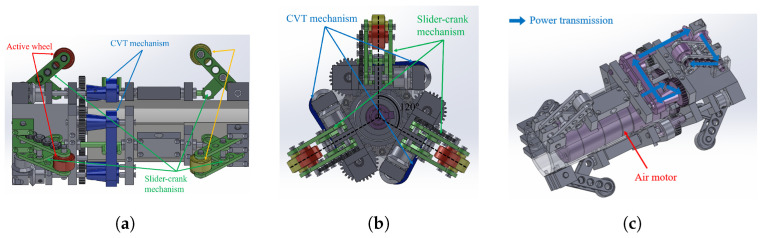
The schematic illustrates the proposed in-pipe robot, showcasing key mechanisms, including six arms with wheels (comprising three active and three passive wheels), three continuous variable transmission (CVT) mechanisms, six slider-crank mechanisms, and a power transmission system powered by an air motor. The active wheels are strategically positioned 120° apart from each other. (**a**) Side view of the the robot; (**b**) front view of the robot; (**c**) power transmission.

In our robot configuration, the front three arms are linked to three active wheels, while the other three arms are connected to three idler wheels at the rear. All active wheels are powered by a single motor located inside the robot. This motor, as illustrated in Figure 2c, drives the active wheels through a gear system and a continuous variable transmission (CVT) mechanism. During the robot’s movement, the wheels on the slider-crank mechanism consistently exert force against the wall, creating traction. The CVT mechanism enables the adjustment of each active wheel’s velocity.

In each slider-crank mechanism, a spring is connected between the slider and the robot’s body to generate a wall-press force from the wheel to the pipe. When the pipe’s diameter changes, the position of the wheel can be adjusted by rotating the crank, a movement induced by the change in the spring’s length. The robot, adaptable to various pipe diameters, can navigate through pipes with changing diameters due to the adjustable spring length. Importantly, since the springs on the sliders are independent of each other, the robot can traverse complex pipe geometries, including curved pipes where the radius from each wheel to the center of the robot differs. The kinematic constraints of the parameters of the slider-crank and the spring for the robot to climb a vertical pipe are presented in Section 2.3.

The velocity of each active wheel can be adjusted using the CVT mechanisms. Specifically, when the robot navigates through a curved pipe, as explained in the preceding paragraph, the wheel’s position changes according to the cross-sectional area’s shape, resulting in a shift in the slider’s position. This movement, in turn, adjusts the belt between the two pulleys of the CVT mechanism. Consequently, the gear ratio of the transmission, associated with the wheel’s speed, undergoes variation. In this configuration, a gear system with a gear ratio of 19:1 was attached to the drive pulley to reduce the wheel’s speed and enhance the transmitted torque value to the gear. Detailed expressions elucidating the speed variation of each wheel using the CVT mechanism will be presented in Section 2.4. It is important to note that, given the independence of the three CVT mechanisms for the active wheels, the speed of each active wheel can be adjusted independently.

**Figure 3:**
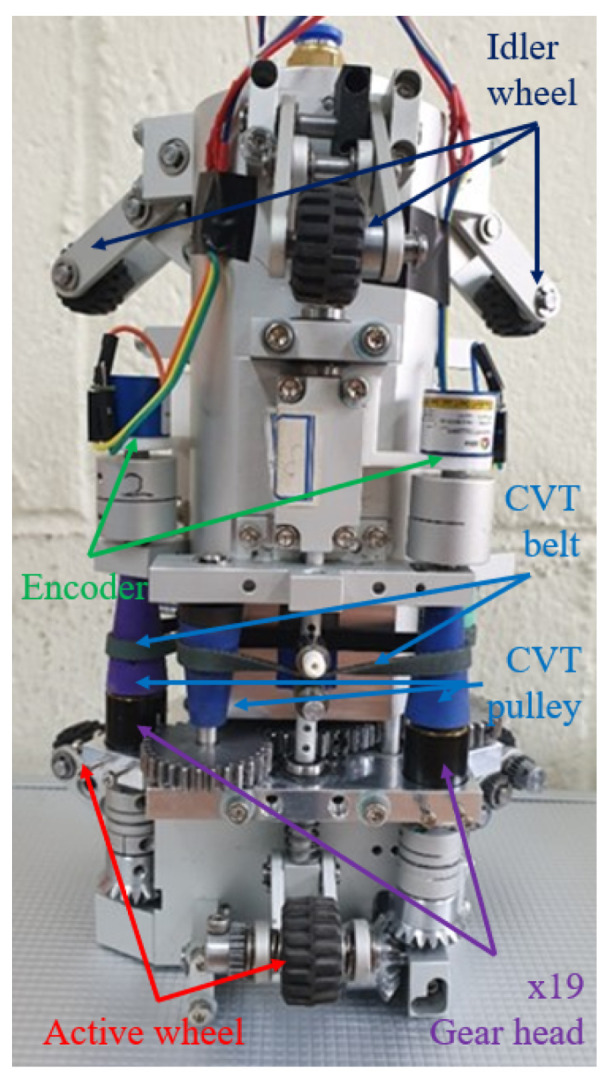
A visual representation of the proposed robot is depicted in the image. The robot features six arms, three of which are connected to idler wheels, and the remaining three are linked to active wheels. The air motor, concealed within the robot’s body, transmits motion to the wheels through a gear and a continuously variable transmission (CVT) system. Notably, the CVT gear is adjusted based on the position of the slider in the slider-crank mechanism, dynamically adapting to the pipe geometry. The rotation of the wheels is recorded using encoders installed at the driven pulleys.

A depiction of the proposed robot is presented in Figure 3. In addition to standard commercial products such as wheels, gear heads, springs, and ball bushings, as well as the air motor, the majority of the robot components—such as the body frame and arms—were fabricated using cutting methods from aluminum materials, following customized 3D design models. The detailed specifications are as follows: the robot is 230 mm in length and can navigate pipes with a diameter ranging from 135 mm (when the robot arm is shortened) to 185 mm (when the robot arm is unfolded). The robot body weighs 1.8 kg, and the air motor contributes 1.5 kg, resulting in a total robot weight of 3.3 kg. Power is transmitted to the air motor via a pneumatic hose connected to the rear of the robot.

### 2.3. Slider-Crank Mechanism

**Figure 4:**
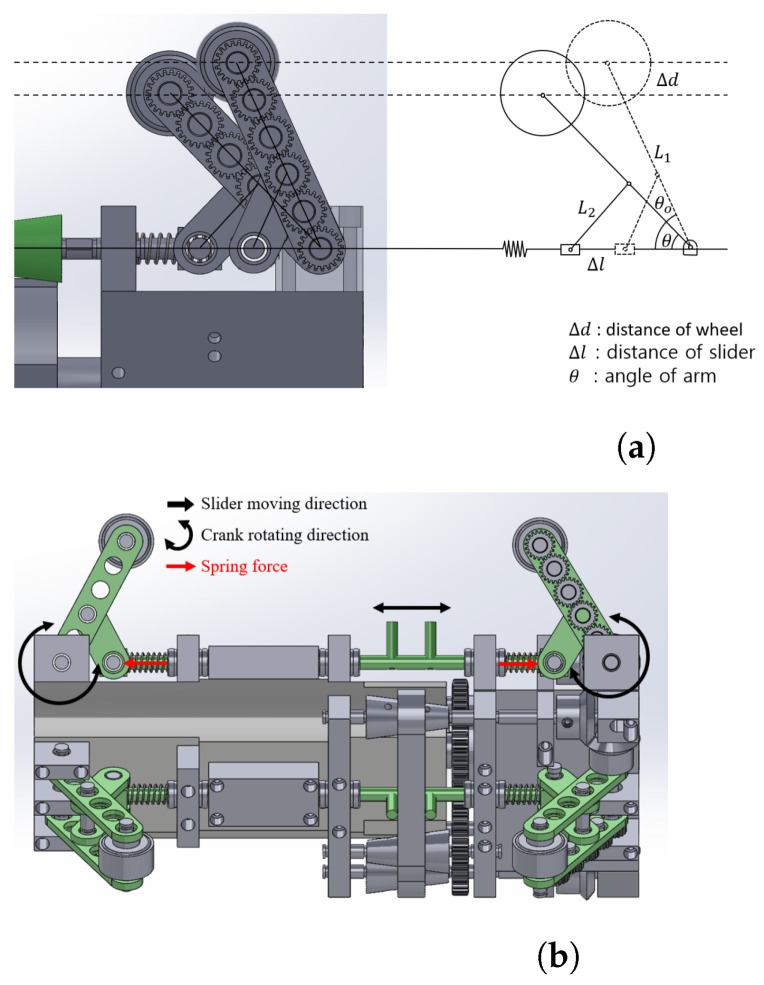
The schematic of the slider-crank mechanism. (**a**) Schematic and kinematic parameters of the slider crank mechanism. (**b**) The zoomed view of the slider crank mechanism. In the zoomed view, two small columns in the middle of the slider play a crucial role in adjusting the position of the CVT belt positioned between them. (**a**) Schematic of slider-crank mechanism; (**b**) a zoomed view of the slider-crank mechanism.

This section explores the constraints on the kinematic parameters of the slider-crank mechanism and the spring, crucial for ensuring adequate traction force during vertical pipe climbing. The kinematic model of the slider-crank mechanism employed in our design is illustrated in Figure 4a.

The total wall press force of the wheels of the robot must satisfy Equation (1):

(1) $$6\mu_{wheel}\cdot F_{n}\geq m_{robot}\cdot g$$

Here $F_{n}$ represents the wall-press force of each wheel, $m_{robot}$ is the mass of the robot, *g* denotes the acceleration due to gravity, and $\mu_{wheel}$ is the coefficient of friction between the wheel and the pipe.

Assuming that the initial angle of the arm is denoted as $\theta_{0}$, and the angle of the arm when the robot enters the pipe is θ. The wheel position change Δd is related to the arm angle by Equation (2).

(2) $$\Delta d=L_{1}\sin\theta_{0}-L_{1}\sin\theta$$

where $L_{1}$ is link length with gear chain.

The corresponding travel distance of the slider Δl is

(3) $$\Delta l=2\cdot (L_{2}\cos\theta -L_{2}\cos\theta_{0})$$

where $L_{2}$ is the other link length. With the link length ratio designed to be $L_{1}$: $L_{2}$ = 5:2, Equation (3) can be also rewritten as follows:

(4) $$\Delta l=2\cdot (\frac{2}{5}L_{1}\cdot \cos\theta_{0}-\frac{2}{5}\sqrt{{L_{1}}^{2}-{\left(L_{1}\sin\theta_{0}-\Delta d\right)}^{2}})$$

Since the travel distance of the slider is the compression distance of the spring, the spring force is

(5) $$F_{s}=k\cdot \Delta l+F_{0}$$

here $F_{0}=k\cdot 0.5$ mm is the spring force caused by the compression of the spring at the initial position. *k* is the spring constant and Δl is the compression distance.

The wall-pressing force $F_{n}$ is related to the spring force by the following equation:

(6) $$F_{n}=F_{s}\cdot tan\theta$$

From Equations (1) and (6), for the robot to climb the vertical pipe the spring stiffness needs to satisfy the condition

(7) $$k\geq \frac{m_{robot}g}{6\mu_{wheel}\left(\Delta l+0.5\right)tan\theta }$$

When the robot passes through a vertical pipe with an inner diameter of 150 mm, the θ is 41.37° and Δl is 15.16 mm calculated based on Equation (4). With the weight of 3.3 kg and the coefficient of friction between the wheel and the pipe of 0.75, from Equation (1) becomes

(8) k≥0.52(N/mm)

Based on the required dimensions and stiffness condition of the spring, a commercial spring from MITSUMI with k=1 N/mm was chosen, which corresponds to a traction force of 62.06 N. The traction force can be made larger by the selection of a stiffer spring (larger stiffness *k*).

### 2.4. Cvt Mechanism

**Figure 5:**
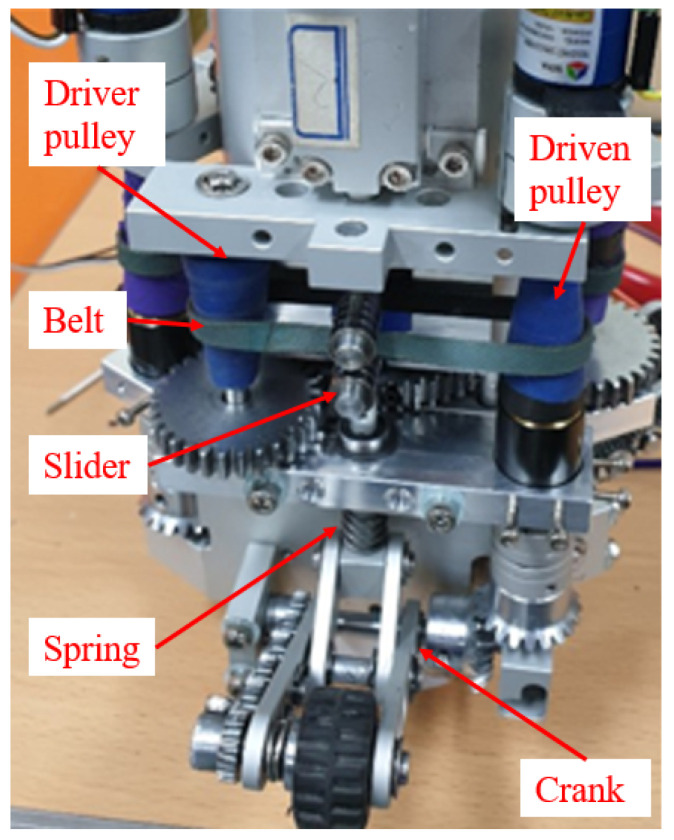
Figure of the CVT mechanism used in the robot. The CVT mechanism includes a driver pulley, a driven pulley, and a belt. The belt’s position is adjusted by the slider in the slider-crank mechanism.

**Figure 6:**
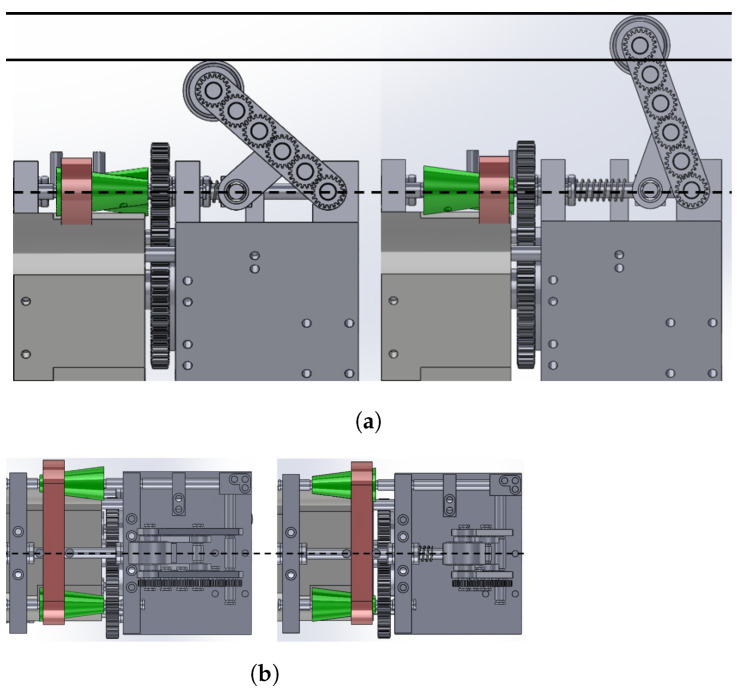
The CVT mechanism serves to adjust the speed of the wheel. In this arrangement, the driver pulley is linked to the air motor, as illustrated in Figure 2c, while the driven pulley is connected to the wheel through a gear system with a ratio of 19:1. The driver pulley and driven pulley are interconnected by a belt. Altering the position of the belt, attributed to changes in the slider’s position influenced by the wheel’s movement, results in a modification of the velocity ratio of the CVT mechanism. This mechanism enables the robot to independently adjust the velocities of its active wheels using only one motor, facilitating navigation through curved pipes. (**a**) Side view of CVT mechanism. The belt’s position is changed from left to right figure; (**b**) top view of CVT mechanism corresponding to the top figure.

As mentioned in Section 2.2, CVT mechanisms were employed to regulate the speed of each active wheel, facilitating the navigation of curved pipes. As shown in Figure 5 and Figure 6, the driver pulley is connected to the motor, while the driven pulley is linked to the wheel. A belt connects the driven pulley and the driver pulley. Altering the position of the belt induces a change in the driven speed. Consequently, the belt’s position is connected to the gear ratio between the two pulleys. This section elucidates the relationship between the driven speed and the position of the belt, given the speed of the driver’s pulley.

**Figure 7:**
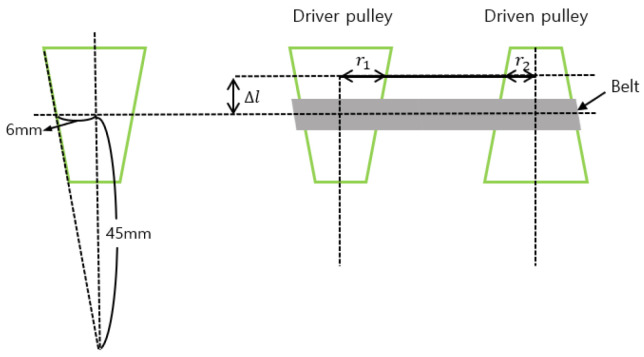
Schematic of CVT’s pulley.

The initial position of the belt is at the center of the pulley, as shown in Figure 7. The height to the center of the pulley is 45 mm and the radius of the center of the pulley is 6 mm.

Since the belt is connected to the slider, the gear ratio of the CVT changes according to the amount of change and can be expressed in the following. We have

(9) $$6:45=r_{1}:\left(45+\Delta l\right)$$

Therefore

(10) $$r_{1}=6\cdot \frac{45+\Delta l}{45}$$

(11) $$r_{2}=6\cdot \frac{45-\Delta l}{45}$$

The speed of the driven pulley is then

(12) $$w_{{driven}_{i}}=\frac{45+\Delta l}{45-\Delta l}\cdot w_{driver}$$

From Equation (4), the speed of the drive pulley becomes

(13) $$w_{{driven}_{i}}=\frac{45+2\cdot (\frac{2}{5}L_{1}\cdot \cos\theta_{0}-\frac{2}{5}\sqrt{{L_{1}}^{2}-{\left(L_{1}\sin\theta_{0}-\Delta d\right)}^{2}})}{45-2\cdot (\frac{2}{5}L_{1}\cdot \cos\theta_{0}-\frac{2}{5}\sqrt{{L_{1}}^{2}-{\left(L_{1}\sin\theta_{0}-\Delta d\right)}^{2}})}\cdot w_{driver}$$

**Figure 8:**
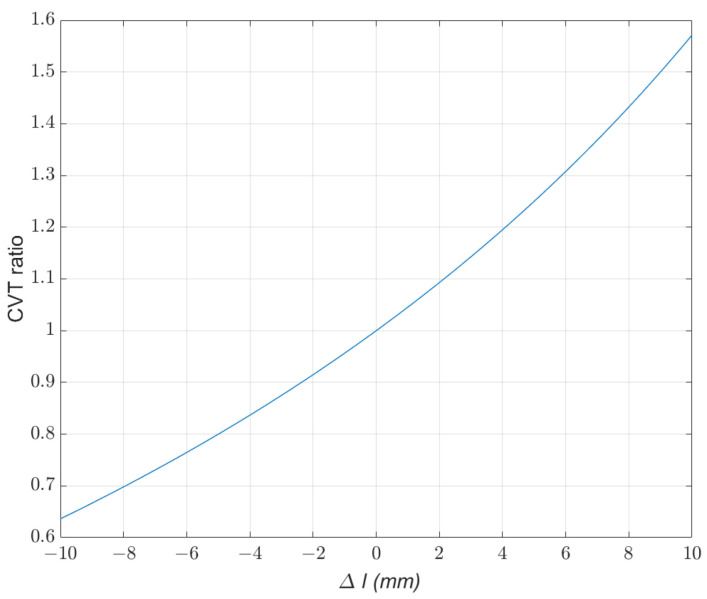
The relationship between the CVT ratio and the compression of the spring is demonstrated in the data, indicating that the CVT ratio can be adjusted using Δl, which is dependent on the position of the wheel relative to the center of the robot.

The relationship between the CVT ratio and the compression of the spring is demonstrated in Figure 8.

## 3. Simulation

### 3.1. Simulation of the Robot Passing through A Curved Pipe

A simulation using MATLAB is conducted to assess the robot’s feasibility of traversing a given pipe before undertaking physical experiments. Evaluating a successful simulation involves verifying the robot’s ability to navigate the pipe without violating its kinematic and contact constraints. Unlike moving through a straight pipe, as illustrated in Figure 1, this becomes less straightforward in a curved pipe. In such cases, the robot needs to independently adjust the speed of each wheel. Simulations are instrumental in evaluating and refining the robot’s design prior to manufacturing. Additionally, we anticipate that this approach will help mitigate the risk of failure or damage during the robot’s deployment in real-field tests.

**Figure 9:**
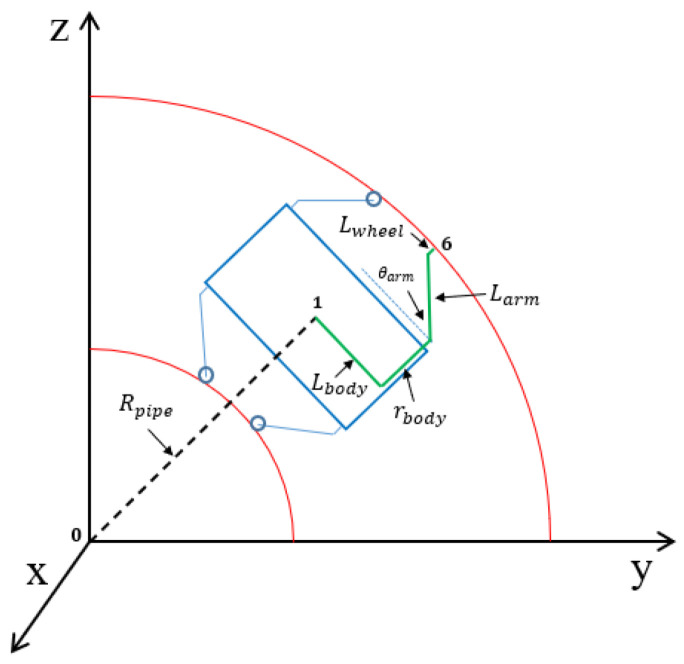
The illustration depicts the position of the robot and the wheel within the pipe. With knowledge of the robot’s position, the wheel’s location can be calculated based on the contact constraint and the kinematics relationships, ensuring that the wheel maintains contact with the pipe during movement.

In this simulation, we assume a constant motor velocity, and the wheels maintain contact with the inner surface of the pipe throughout the movement. Initially, the robot’s position, orientation, and the motor’s velocity are specified. Utilizing the kinematics model based on the Denavit–Hartenberg (DH) method, which relates the positions and orientations of the wheels and the robot, along with the spring compression and wall-press force models developed in Section 2.3, and the CVT gear ratio model in Equation (13), we calculate the next positions and orientations of the robot and wheels that satisfy the constraints. The position of the robot and the wheel within the pipe is illustrated in Figure 9.

**Figure 10:**
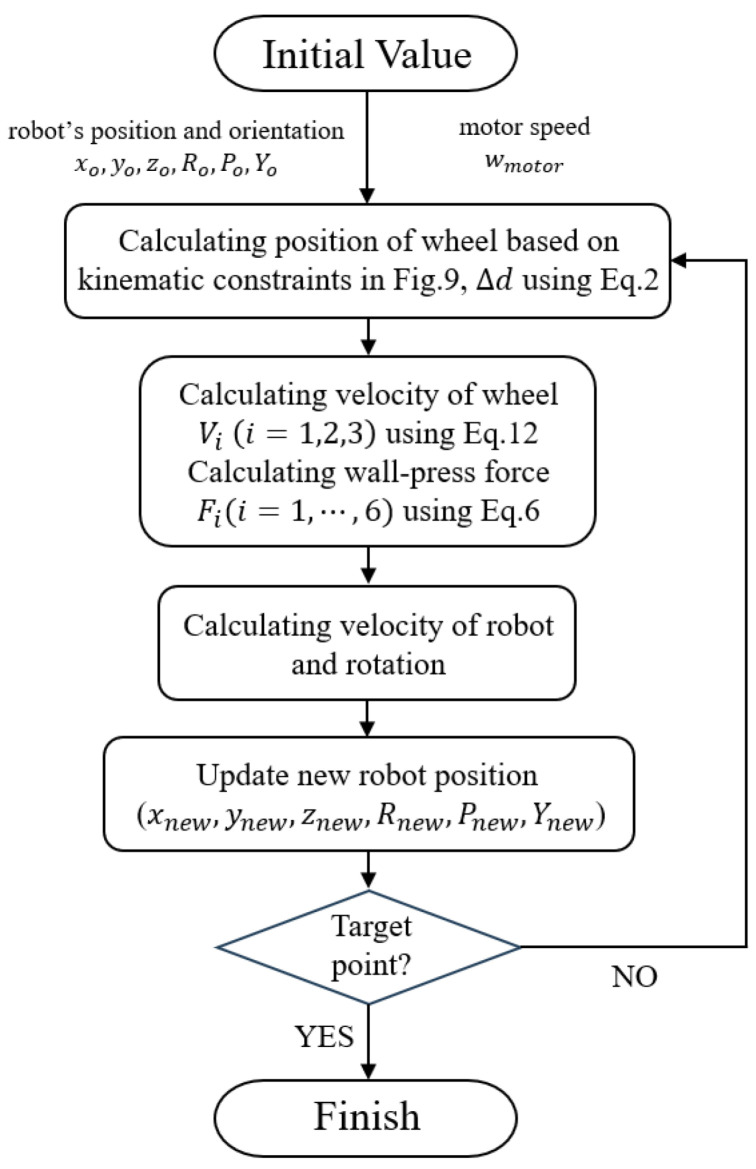
Flow chart of the simulation process.

It is noteworthy that, in addition to the wall-contact constraint, the wheel’s velocity must adhere to the constraint described in Section 2.1. This constraint stipulates that the rotational velocity of the wheel with a larger turning radius should be greater. The simulation is considered successful if the robot can navigate the curved pipe without violating any constraints. The simulation process can be seen in Figure 10.

**Figure 11:**
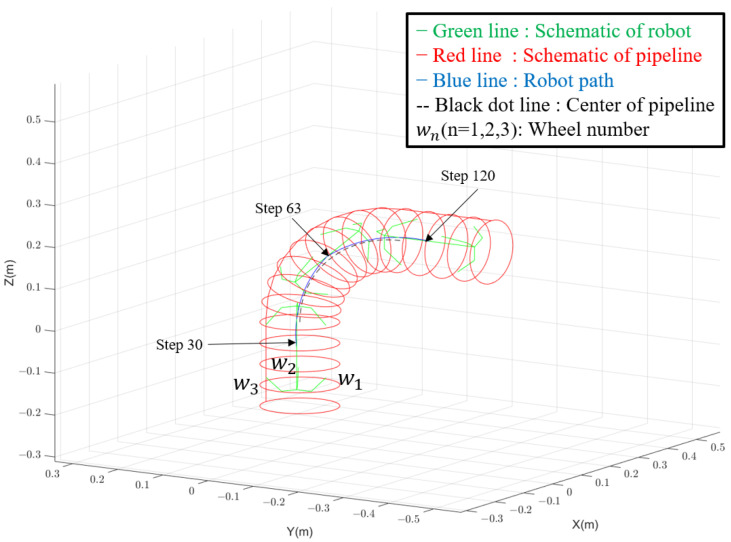
The schematic of the pipeline and the illustration of the simulation are presented. In each time step, the robot calculates the wheel’s position, velocity, and wall-press force based on its current position and orientation, ensuring compliance with kinematic constraints. The blue line shows the successful path of the robot moving inside the curved pipe without violating kinematic constraints, which verify the design.

The above process is shown in the following Figure 11.

**Figure 12:**
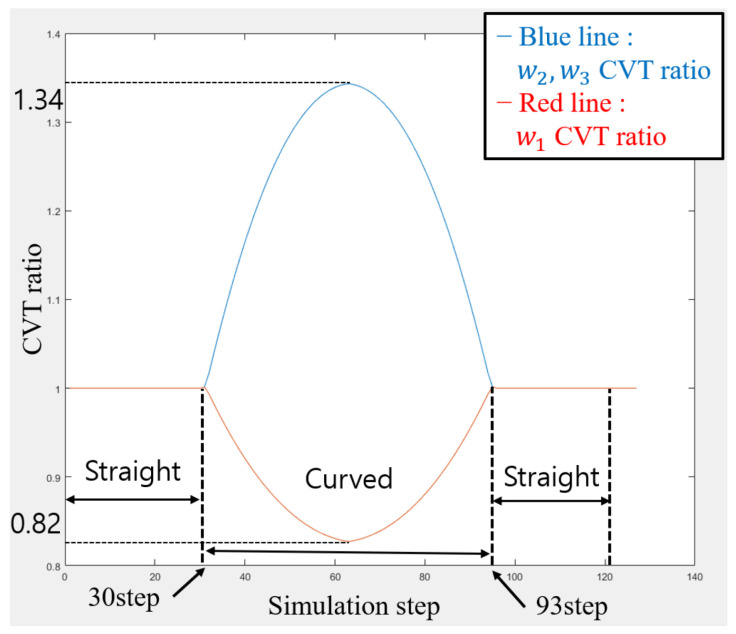
The velocity of the wheels during movement in Figure 11. It is seen that the speeds of all wheels are uniform and constant when navigating straight pipes. However, in curved sections, the speed of Wheel 1, positioned closer to the curve turning center, is slower compared to Wheels 2 and 3.

The simulation results are seen in Figure 11 and Figure 12. The simulation results seen from Figure 12 demonstrate the robot’s successful traversal through straight-curved-straight pipes without violating kinematic constraints. In straight sections, all wheels maintain a uniform and constant speed. However, during the curved pipe passage from step 30 to step 93, wheel 1, positioned closer to the curve turning center, exhibits a slower speed compared to wheels 2 and 3. This discrepancy is attributed to changes in the wheel distance, Δd, as the robot transitions from the straight pipe to the curved pipe, influencing the speed.

### 3.2. Minimum Radius of the Curved Pipe

**Figure 13:**
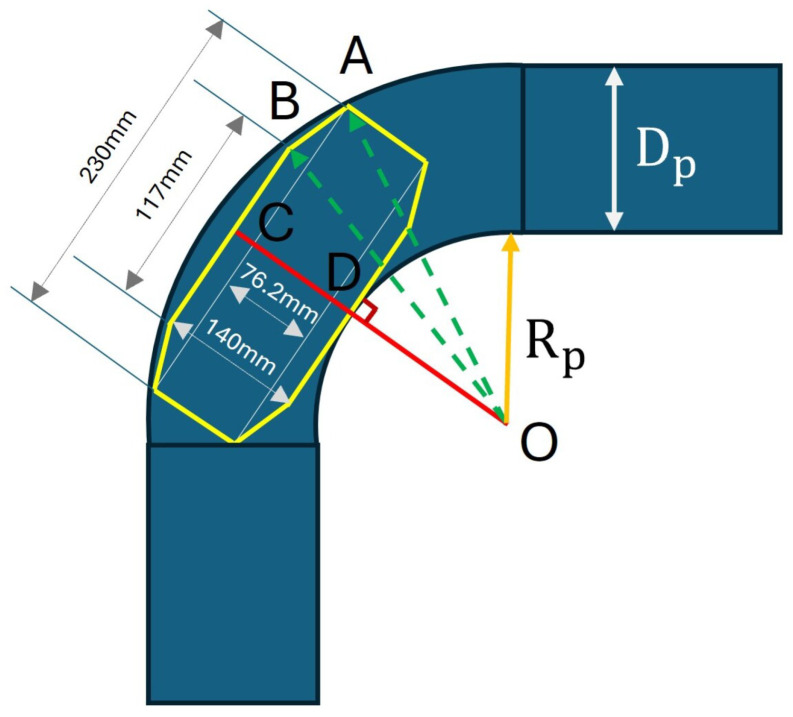
Illustration of the robot inside a curved pipe section. The yellow frame shows the robot’s boundary when the arm is at the position where the CVT mechanism can start to function. The robot will be stuck inside the pipe in two cases: due to its length (leg at B is adjustable but A is stopped by the pipe’s surface) or due to its width (B is stopped by the pipe’s surface and the CVT mechanism does not function normally).

The minimum sizes of the curved pipes that the robot can pass through can be calculated based on Figure 13. In this context, the diameter of the pipe ($D_{p}$), and the size of the robot, represented by the yellow frame in Figure 13, are known. The robot’s frame is defined as the boundary of the robot when the arm is at a position where the CVT mechanism can function normally. The challenge is to determine the minimum curve radius ($R_{p}$) necessary for the robot to navigate the curve.

The robot may encounter two scenarios leading to it getting stuck inside the pipe: first, due to its length (case 1, where the leg at B is adjustable but A is stopped by the pipe’s surface), or second, due to its width (case 2, where B is stuck at the pipe’s surface, and the CVT mechanism does not function normally). The minimum values of $R_{p}$ in these cases can be easily determined as follows.

(14) $${\left(R_{p}+105.06\right)}^{2}+115^{2}=OA^{2}={\left(R_{p}+D_{p}\right)}^{2}\;\left(Case1\right)$$

(15) $${\left(R_{p}+140\right)}^{2}+{\left(117/2\right)}^{2}=OB^{2}={\left(R_{p}+D_{p}\right)}^{2}\;\left(Case2\right)$$

The minimum value of $R_{p}$ so that the robot can pass through the curved pipe with a radius of $D_{p}=150$ mm is the larger one between the $R_{p}$ values obtained from Equations (14) and (15), which is 26.11 mm.

## 4. Experiments

To validate the performance of the robot design, experiments were conducted to showcase its ability to climb vertical, straight, and curved pipes. The proposed in-pipe robot is depicted in Figure 3. The air motor used for the robot is AMV-050 model from Actosys, and the air pump is a ZN-6050 model from Woojin. The specifications of the air motor and the air pump are listed in Table 3 and Table 4, respectively. Details of the robot’s specifications are also available in Table 5. To measure wheel velocity, rotary encoders (SME360CP-05, SERA) with a resolution of 7200 were installed at the driven pulleys of the CVT mechanisms. The velocity ratio between the driven pulley and the wheel is 19:1, allowing the calculation of wheel velocity. Acrylic pipes with a 150 mm diameter were used in all experiments, consisting of a 1 m straight pipe and a curved pipe with a 0.225 m radius. Experiments 1 and 2 involved a single straight pipe in a vertical configuration, while experiment 3 combined two straight pipes with a curved pipe. Throughout the experiments, the air pump was set at 6 bar, and the air motor operated at its maximum speed corresponding to the maximum flow rate from the pump. The following experiments have been tested.

- Experiment 1: payload measurement. The focus of this experiment is on measuring the payload capability of the robot during vertical pipe climbing. The data collected in this experiment will also serve to validate the traction force target outlined in Section 2.3.
- Experiment 2: vertical pipe climbing. In Experiment 2, the objective is to validate the effectiveness of the slider-crank mechanism design and the spring parameters in generating sufficient force to counteract the weight of the robot during vertical pipe climbing.
- Experiment 3: passing through the curved pipe. Experiment 3 is focused on validating the robot’s capability to navigate through a curved pipe. This experiment aims to confirm that each wheel can independently adjust its velocity, a crucial requirement for successful traversal through curved pipes.

### 4.1. Payload Measurement Experiment

**Figure 14:**
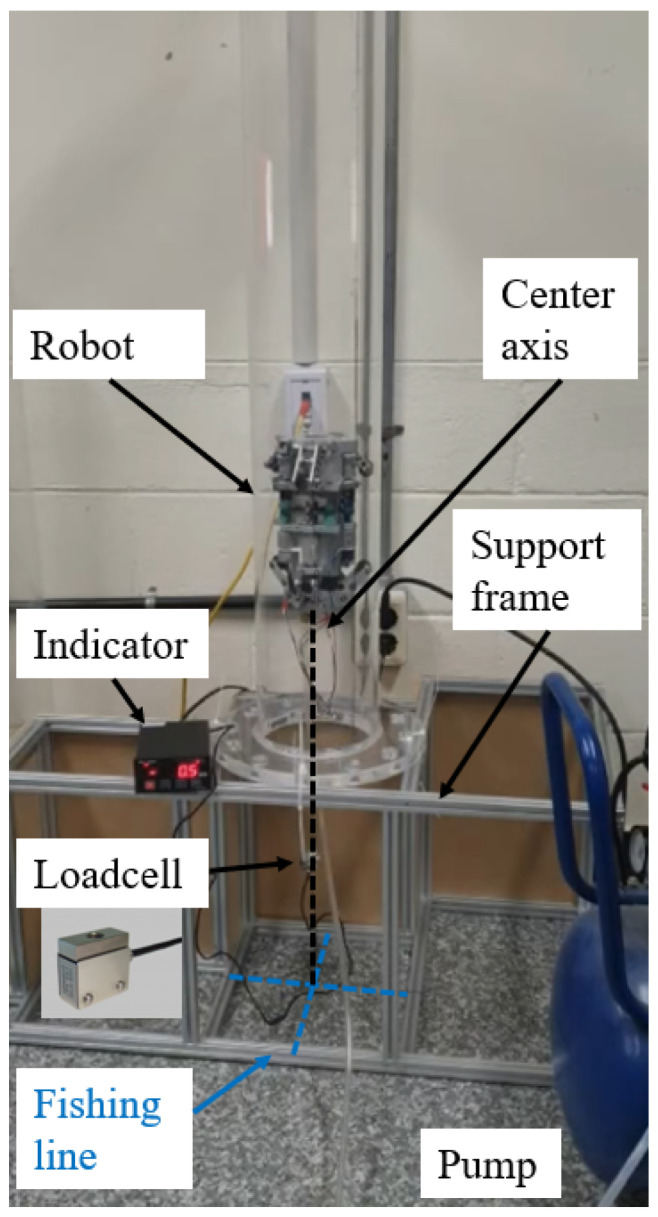
Setting for payload experiment. The payload experiment involves measuring the load using a load cell connected to the rear of the robot via a fishing line. The load cell is also tethered to the experimental frame using fishing lines, ensuring that the position of the fishing line remains at the center of the pipe as the robot climbs.

In this experiment, the payload capability of the robot will be measured to validate the results obtained through calculations in Section 2.3. A vertically erected straight pipe is utilized, and the robot is driven inside the pipe. As illustrated in Figure 14, a load cell (UU3 from DACELL) is connected by a line to the center of the rear of the robot. The load cell is connected to the frame using fishing lines (depicted by the blue dotted line).

Eight experiments were conducted, and the mean value was obtained. The results demonstrate that the robot can successfully climb a vertical pipe, with a measured payload capability of 24.5 N when the robot pulls the fishing line. The force value increased to 28.42 N due to an increase in the pulling force of the line before the robot experienced a slight descent.

The traction force generated by the robot can be calculated at the minimum pulling value of 24.5 N. Considering the gravitational force caused by the weight of the robot as 3.3 kg × 9.8 = 32.34 N and the pulling force as 24.5 N, the traction force of the robot is 32.34 N + 24.5 N = 56.84 N. This experimental value closely aligns with the theoretical design value of 62.06 N discussed in Section 2.3.

The maximum travel distance $l_{max}$ of the robot working on a straight pipe can be calculated as follows. Assuming that the main load comes from the total weight of the pneumatic hose that the robot carries. Since our hose weights 8 g/1 m, the maximum weight that the robot can carry is $8l_{max}\left(g\right)$, which results in a load value of $8\times 9.81l_{max}/1000=0.0785l_{max}N$ in a straight vertical pipe case. For the maximum pulling force of 24.5, the maximum travel distance of the robot in a vertical pipe is then $l_{max}=24.5/0.0785=312.5$ m. In the horizontal pipe, $l_{max}=312.5$ m/0.8 = 390.6250 m, where 0.8 is the coefficient between the hose (dry rubber) and the pipe (acrylic). In reality, the maximum travel distance of the robot will be smaller than the calculated value due to many reasons such as wire vibration. The maximum travel distance of the robot inside a pipeline with various pipe directions and geometries is also expected to be smaller than the calculated values, which might be also estimated if the pipeline’s design is known. The limited travel distance might be addressed using a portable pressurized tank, like the self-power solution in [31]. That will be a topic to be considered in our future work.

### 4.2. Vertical Pipe Climbing

The setup for this experiment is similar to that in Section 4.1. However, in this case, the load cell setup is not used. This experiment aims to explore the robot’s capability to climb a vertical pipe and to measure its speed, which is calculated from the encoder values.

**Figure 15:**
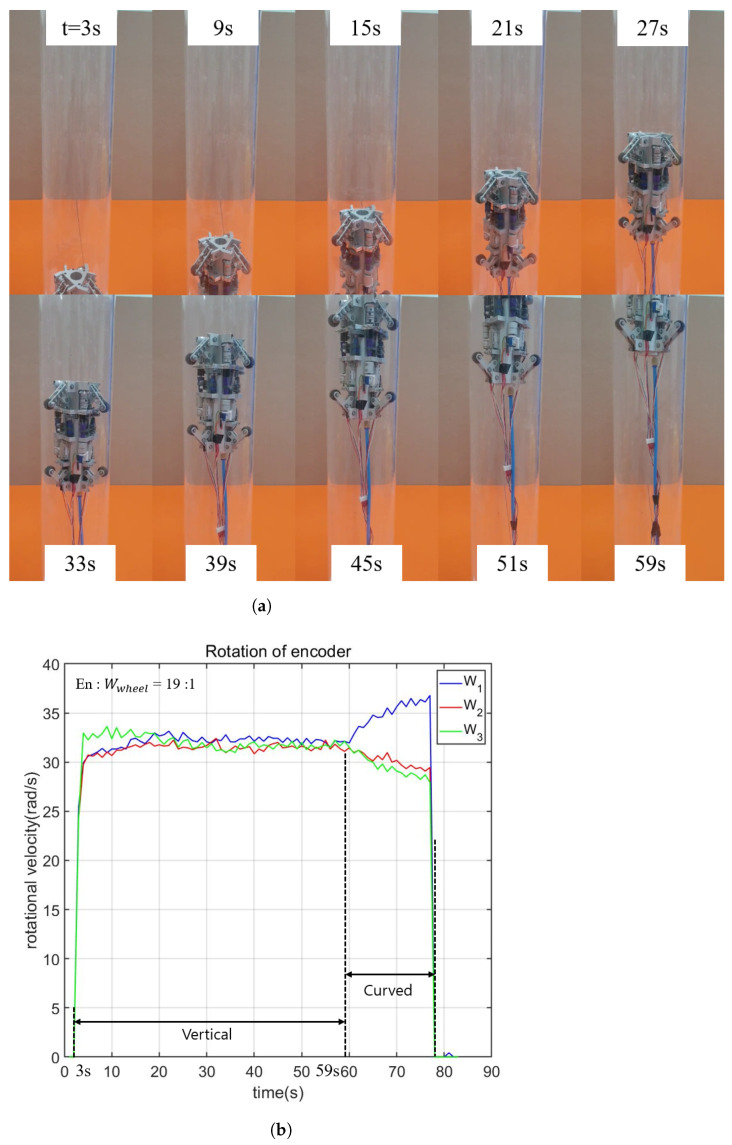
Vertical pipe climbing experiment. (**a**) snapshots of the robot since the robot enters the pipe at 3s until the robot climbs 96.045 mm up to the pipe at 59 s. (**b**) Angular velocities of driven pullies calculated from the encoder values. The velocities of the three driven pullies are similar when the robot is at a steady state from 10 s to 59 s. From 3 s to 10 s, due to the differences in the initial conditions between wheels, the velocities of the pullies are different from those after 10 s. After the second 59, the robot successfully climbs up the vertical pipe and moves to the connected curved pipe, which is not considered in this experiment. (**a**) Snapshots of the robot climbing a vertical pipe; (**b**) Angular velocities of driven pullies calculated from the encoder values.

The snapshots of the experiment are shown in Figure 15a. The video demonstrating the vertical pipe experiment can be found in the Supplementary Materials (Videos S1 and S2). It is seen that the robot successfully climbs the vertical pipe with a length of 96.045 mm in 56 s (from 3 s to 59 s). The rotation speeds of three active wheels, which are calculated based on the encoder values considering the pulley-to-wheel speed ratio, are shown in Figure 3. During the steady state, the rotational velocities of the three driven pulleys are nearly identical, with an average value of 32±2 rad/s. Considering the deceleration ratio of 1/19, the corresponding rotational speed of the wheel is approximately 1.685 rad/s. Given the wheel’s radius of 10 mm, the robot’s speed is estimated to be about 1.685 mm/s. It is worth noting that from the time the robot enters the pipe at 3 s until 10 s, slight differences in the speeds of the driven pulleys and each driven pulley’s speed compared to those at the steady state are observed. This discrepancy may arise from variations in the initial states of the wheels when the robot is initially forced into the pipe.

### 4.3. Curved Pipe Driving

In this experiment, the robot traverses a combination of straight-curved-straight pipes in a horizontal setting. Similar to the vertical climbing case, the speed of each wheel is calculated based on the encoder values recorded during movement. As discussed in Section 2.2, the robot must be capable of adjusting each wheel independently to navigate through a curved pipe. This is because the wheel with a larger turning radius needs to move at a faster velocity compared to the one with a smaller turning radius.

**Figure 16:**
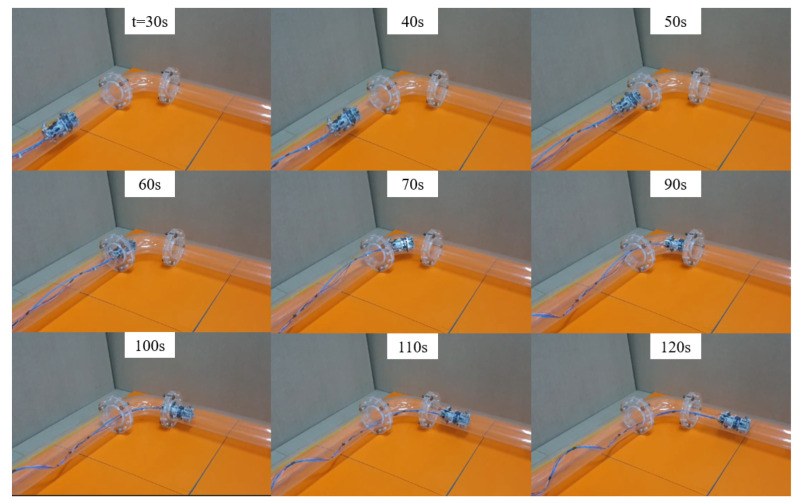
Curved pipe passing experiment. The experiment shows that the robot can successfully pass a combination of straight-curved-straight pipes.

**Figure 17:**
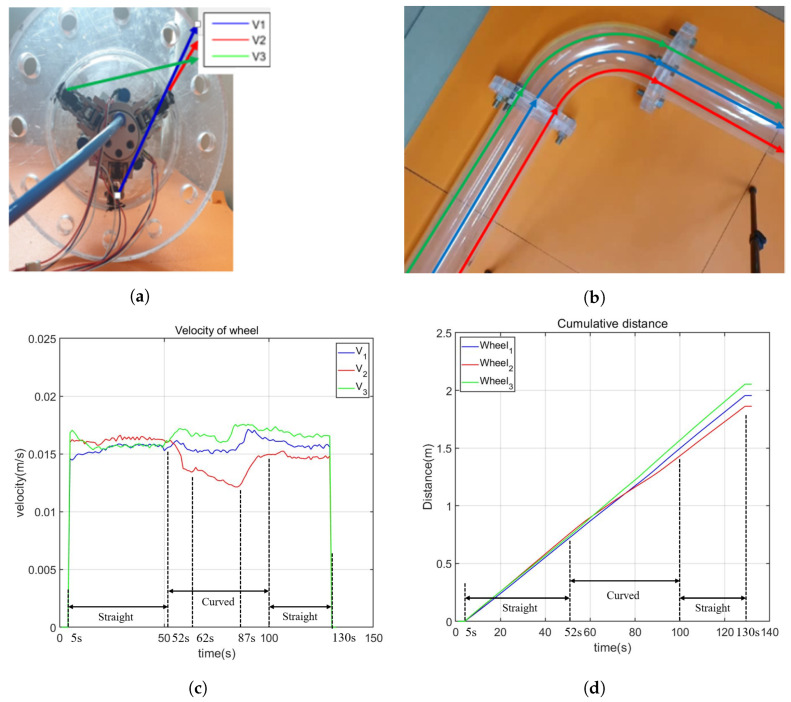
First curved pipe passing experiment. (**a**) Positions of the wheels. (**b**) Expected path of the wheels. (**c**) Velocity of the wheels. The velocities of all the wheels are similar when the robot moves inside the straight pipe. From the second 52 s, the robot enters the curved pipe, and the velocities of the three active wheels differ. The wheel with a larger turning radius has a higher velocity until the robot successfully exits the curved pipe at the second 100. It is noteworthy that the second 61 marks the moment when the robot fully enters the curved pipe, while the second 87 indicates the start of the robot entering the second straight pipe. (**d**) The moving distance of the wheels. Before the second 52, the moving distance of all the wheels is nearly the same, but they become different when the robot enters the curved pipe due to the varying turning radii. (**a**) Initial positions of the wheels; (**b**) path illustration of each wheel; (**c**) velocity of the wheels; (**d**) distance of the wheels.

The first experimental results can be seen in the Figure 16 and Figure 17, in which, the snapshots of the experiment are shown in Figure 16. The video demonstrating the curved pipe experiment can be found in the Supplementary Materials (Video S3). The positions of the wheels are arranged as in Figure 17a, and the expected path of each wheel is illustrated in Figure 17b. Figure 17c,d shows the velocities of the wheels and their moving distance inside the pipe. From Figure 17c, it is observed that the velocities of all the active wheels are nearly the same with an average value of 0.016 mm/s when moving in the straight pipe. During the period when the robot enters the curved pipe until it moves out the curved pipe from 54 s to 109 s, the velocities of the 3 wheels are different, which are caused by the different turning radii. The differences in the turning radius are verified by the moving distances of the wheels shown in Figure 17d. It is observed that the average speed of the wheels is 0.0178 mm/s, 0.0151 mm/s, and 0.0136 mm/s, in which the velocity order is the same as the moving distance of each wheel. The successful curved pipe passing of the robot therefore verified the proposed robot structure using the cvt mechanism to vary the wheel’s speed adaptive to the pipe geometry.

**Figure 18:**
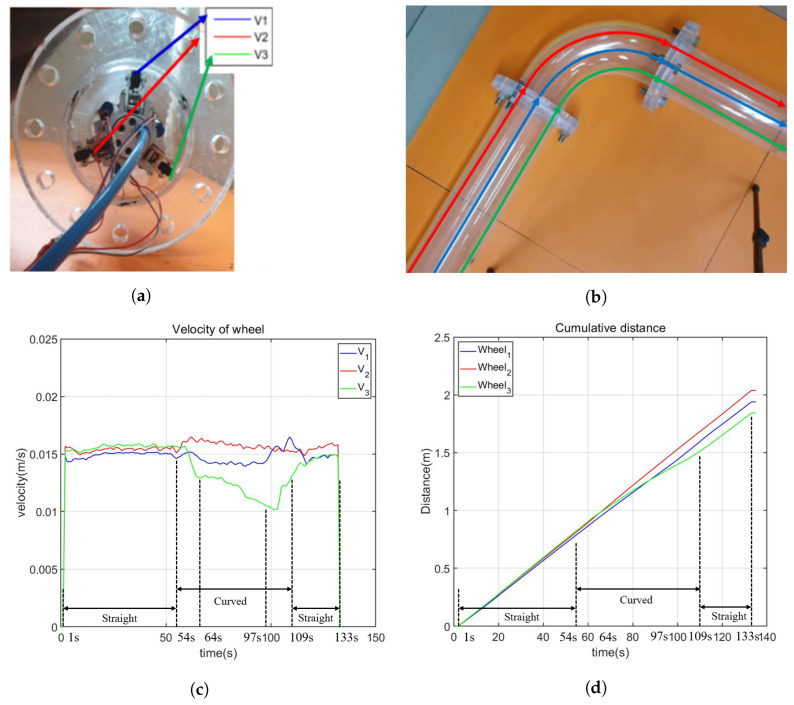
Second curved pipe passing experiment. (**a**) Positions of the wheels. The current configuration is symmetric to the configuration in (**b**) Expected path of the wheels. (**c**) Velocity of the wheels. The velocities of all the wheels are similar when the robot moves inside the straight pipe. From the second 54s, the robot moves into the curved pipe. Similar to the results shown in Figure 17, the velocities of 3 active wheels are different, in which the wheel with a larger turning radius has a larger velocity until the robot successfully passes the curved pipe at the second 109. Here, the second 64 is the moment when the robot completely moves inside the curved pipe, and the second 97 is the moment when the robot starts to enter the second straight pipe. (**d**) The moving distance of the wheels. Before the second 54, the moving distance of all the wheels is nearly the same, but they become different when the robot moves into the curved pipe since the turning radii are different. (**a**) Initial positions of the wheels; (**b**) path illustration of each wheel; (**c**) velocity of the wheels; (**d**) distance of wheel.

Furthermore, to assess the impact of the robot’s configuration on its performance, we conducted a variation of the previous experiment by rotating the robot 180 degrees. This adjustment resulted in a symmetric robot configuration about the horizontal plane through the axis of the pipe. The experimental setup and results are depicted in Figure 18. Similar behavior was observed in the robot compared to the previous experiment. Specifically, the velocity of all wheels remained consistent at 0.015 mm/s when the robot moved in the straight pipe, and the wheel with a larger turning radius exhibited a higher velocity than the one with a smaller turning radius. However, it is important to note that the robot’s performance in this experiment showed slight variations compared to the previous one. There were differences in the velocities of wheels with the same turning radius, and the time taken by the robot to enter and complete the curved pipe also differed. In the initial experiment, the robot took 48 s (from second 52 to second 100) to pass through the curved pipe, while in this second experiment, it took 55 s (from second 54 to second 109). We attribute these variations to potential nonlinear effects induced by the weight of the robot. Addressing these differences and developing a dynamic model for more accurate motion prediction will be considered in our future work.

## 5. Conclusions and Future Work

In this paper, we developed a wheel-type in-pipe robot capable of climbing both vertical and curved pipes. The vertical climbing ability is facilitated by a slider-crank mechanism with a wall-push force generated by a spring connected to the slider. The variable velocity of each active wheel is achieved through a CVT mechanism, whose speed ratio is adjusted by the position of a belt connected to the slider of the slider-crank mechanism. The adaptive change in the position of the slider, corresponding to the geometry of the pipe, allows for independent variation in the velocity of each active wheel. This feature aids the robot in successfully navigating through curved pipes using only one motor. Our approach was validated through both simulations and experiments.

A comparison between our robot and other similar robots can be seen in Table 6, in which various in-pipe robots with different locomotion types (wheel, Caterpillar, Inchworm) and actuators (electric motors, pneumatic motors, SMAs) were compared. One weakness of our robot compared to other robots is its limited capability of overcoming various types of pipes, which comes from its structure. One identified weakness of our robot, in contrast to other models, is its limited capability to overcome various types of pipes due to its current single-module structure. This limitation prevents effective navigation through complex pipes, such as T-branches or miter pipes. However, we plan to address this issue in future work by developing additional modules. On the positive side, a notable advantage of our robot lies in its passive velocity control method, utilizing CVT and slider-crank mechanisms. This feature enables the robot to use only a single actuator for achieving independent velocity control for each wheel without prior knowledge of the geometry of current pipe section. Additionally, this capability allows for the minimization of electronic components on the robot. In fact, our robot operates without any on-board electronic parts (encoders were only used for data collection purposes). This design not only enhances endurance in extreme environments, such as high temperatures, but also makes the robot suitable for operation in explosive environments through the use of an air motor, as explained in the following.

It should be mentioned here that the selection of a motor for our robot is influenced by the specific target application in our project. In this context, the current module marks the initial step towards designing a robot capable of safely cleaning and inspecting the inside of a chemical pipeline system in a semiconductor factory. The atmosphere within the pipeline has the potential to consist of a mixture of air and flammable substances, including gases and dust that can be combustible. The explosive atmosphere, also known as ATEX (EXplosive ATmospheres), can lead to explosions in the presence of an ignition source caused by excessively high temperatures, sparks, or dust. Among the various types of actuators commonly used for in-pipe robots as introduced in Section 1, artificial muscles such as SMAs are unsuitable as they cannot provide sufficient force and are slow. Electric motors, despite their advantages of precise control and portability, pose a high risk of overheating and sparks [33]. Regarding air motors, a type of pneumatic actuator, since air serves as the working fluid and torque is produced mechanically rather than electrically, air motors are non-sparking. Hence, they are inherently safer for use in hazardous environments, such as ATEX environments, compared to electric motors. This is the primary reason for choosing an air motor in our application. As another potential direction, we believe that the proposed robot with air motors can offer advantages when operating in other hazardous environments, such as those with high temperatures that adversely affect electric motors [34]. As the compressed air power source moves through the motor, it expands and naturally cools. Consequently, air motors are not prone to overheating, allowing them to be deployed in high ambient temperatures up to 150 °C. The deployment of the robot in the described real-world industrial settings will be addressed in our future work.

However, the utilization of air motors comes with a practical drawback, which is a necessity for an air supply reservoir. Increasing the power of the air supply leads to an escalation in the overall system cost. The incorporation of CVT holds significant potential in mitigating power consumption in in-pipe robots, as it allows for a reduction in the number of required actuators. Additionally, as active control of each wheel becomes unnecessary, the complexities of the robot controller can be significantly minimized. Moreover, since active control of each wheel is not needed, the complexities of the robot controller can be significantly reduced. It should be noted here that the first module was designed for the target pipe size of 150 mm, as utilized in our project, and our primary objective was not to maximize its size adaptability range. Consequently, the robot can currently adapt to a pipe range of only 50 mm (135 mm to 185 mm), which is comparatively smaller than the adaptability range presented in [35]. However, this limitation may be mitigated by adjusting certain parameters in future iterations, such as the length of the arm or the stiffness of the spring.

The authors acknowledge certain limitations of the robot that require attention in future work. As stated in Section 2.1, the current version of the robot does not consider the presence of the fluid flow; therefore, it cannot work in the in-service networks. However, we hope to overcome the challenge through design improvements. Specifically, the inclusion of proper sealing will be essential to ensure the normal function of critical components such as gears, slider-crank mechanisms, CVT mechanisms, and the air motor. Additionally, we plan to conduct Computational Fluid Dynamics (CFD) computations, similar to those in [27], to identify the maximum traction force. This information will guide the appropriate selection of the spring’s stiffness, as in Section 2.3, to enhance the robot’s performance. In our upcoming work, we also plan to explore wireless communication and implement simultaneous localization and mapping (SLAM) solutions. Based on our previous work [36] in which several PSD sensors in each module of the in-pipe robot have been used to estimate the pose of the robot and the pipe shape, one possible SLAM method might be the fusion of PSD sensors and odometry data from encoders such as the work in [37]. Here, the odometry data will be used to estimate the travel distance of the robot. However, other localization approaches of the in-pipe robot such as the use of Kalman Filters [38,39,40,41,42], wireless communication [43], acoustic echo-based localization [44], and other SLAM methods [45,46,47,48,49] might be adapted to our future work. Moreover, to facilitate long-distance inspection, the smart motion of the in-pipe robot, which combines the localization and motion controller of the robot such as in [27,35], will also be considered in our next step.

## Figures and Tables

**Table 2:** Comparison of different in-pipe robots (adapted from [25]).

Performance Indicator	Wheel Type			Caterpillar Type		Without Wheel Type				PIG
	**Simple** **Structure**	**Wall-** **Press**	**Screw-** **Drive**	**Simple** **Structure**	**Wall-** **Press**	**Snake**	**Inchworm**	**Legged**	**Free** **Swimming**	
Vertical Locomotion	×	⋎	⋎	×	⋎⋎	×	⋎	×	×	×
Maneuverability	⋎⋎	⋎	⋎	×	⋎	⋎⋎	×	⋎	⋎⋎	×
Variable Diameter Adaptability	×	⋎⋎	⋎	×	⋎⋎	⋎	⋎	×	×	⋎
Motion Efficiency	×	⋎	⋎	⋎	⋎⋎	×	⋎	×	⋎	×
Interference to Flow	⋎	⋎⋎	⋎	⋎	×	××	××	×	⋎	⋎

**Table 3:** Specifications of the air motor.

Output power (W)	281
Max. Torque (N.m)	26.77
Max. Speed (RPM)	180
Max. Air consumption (m^3^/min)	0.22
Weight (KG)	1.1
Size (D, L)	55 mm, 108 mm

**Table 4:** Specifications of the air pump.

Horsepower (HP)	6
R.P.M	1640
Flow rate (l/min)	230
Pressure (BAR)	6
Capacity (l)	50
Noise (DB)	72
Weight (kg)	63
Size (l, w, h)	67 mm, 39 mm, 80 mm

**Table 5:** Specification of the robot.

Minimum diameter (mm)	135
Maximum diameter (mm)	185
Length (mm)	230
Weight (kg)	3.3
Actuator	Air-motor 25.12 Nm
Maximum speed	0.03 m/s
Maximum payload	56.84 N

**Table 6:** Comparison of the proposed robot with other in-pipe robots.

Ref (Type)	Capability (Pipe Types)	Dia.Range (mm) Length (mm) Weigh (kg)	Max.Speed (mm/s) Traction Force (N)	Velocity -Control -Method	Actuator Active Driven -Parts	On board -Electronics
Our robot (Wheel)	Horizontal, vertical, curved	135–180 mm	30 mm/s	passive	1 air motor	no
		230 mm	56.84 N	(CVT	3 wheels	
		3.3 kg		slider-crank)		
[8] (Wheel)	Horizontal, vertical, T-branch	116–127 mm	80 mm/s	active	5 electric	E-motor
		51 mm	202 N	(wheel control)	-motors	circuit board
		2.37 kg			5 wheels	
[9] (Wheel)	Horizontal, vertical	198–305 mm	N/A	active	3 electric	E-motor
		N/A	N/A	(wheel control)	-motor	E-magnet
		1.03 kg			3 wheels	
[32] (Wheel)	Elbow, horizontal, vertical, T-branch	85–109 mm	150 mm/s	active	3 electric	E-motor
		150 mm	9.8 N	(differential	-motors	camera
		0.7 kg		-drive)	2×3 wheels	
[11] (Screw)	Vertical, bent	100–129 mm	500 mm/s	active	2 electric	E-motor
		175.8 mm	N/A	(angle	-motors	
		0.7 kg		-control)	3 wheels	
[6] (Caterpillar)	Vertical, inclined, bent	N/A	260 mm/s	active	1 electric	E-motor
		84 mm	N/A	(caterpillar	-motor, 3 SMAs	
		0.3 kg		-control)	2 caterpillars	
[12] (Caterpillar)	Bent pipes and T-branch	480–650 mm	24.17 mm/s	active	4 electric	E-motor
		1995 mm	324 N	(caterpillar	-motors	circuit board
		70.1 kg		-control)	4 caterpillars	sensor
[18] (Caterpillar)						E-motor
		950–1200 mm	88.3 mm/s	active	3 electric	E-putter
	Horizontal	N/A	N/A	(caterpillar	-motors	camera
		45.8kg		-control)	3 caterpillars	ladar
						pressure sensor
[19] (Caterpillar)	Elbows, T-branches, horizontal, vertical	80–100 mm	90 mm/s	active	3 electric	E-motor
		78 mm	1.18 N	(caterpillar	-motors	camera
		0.266 kg		-control)	3 caterpillars	
[3] (Inchworm)	Vertical, Curved with U-turn	50–150 mm	24.6 mm/s	passive	pneumatic	no
		1000 mm	16–37 N	(pressurized	-actuator	
		N/A		-unit)	3 segment	
[21] (Leg)	Two horizontal plates	30 mm	50 mm/s	active	1 electric	E-motor
		30 mm	N/A	(using leg)	-motor	
		0.324 kg			2 legs	
[22] (Leg)	No in-pipe test	65–100 mm	6 mm/s	active	2 electric	E-motor
		33.06 mm	N/A	(using leg)	-motors	circuit board
		0.0945 kg			6 × 1 set leg	

## Data Availability

Data are contained within the article.

## Supplementary Materials

The following supporting information can be downloaded at: https://www.mdpi.com/article/10.3390/biomimetics9020113/s1, Video S1: Vertical pipe experiment; Video S2: Various diameter pipe experiment; Video S3: Curve pipe experiment.
